# In Vivo Deformation and Strain Measurements in Human Bone Using Digital Volume Correlation (DVC) and 3T Clinical MRI

**DOI:** 10.3390/ma13235354

**Published:** 2020-11-25

**Authors:** Saman Tavana, Jeffrey N. Clark, Nicolas Newell, James D. Calder, Ulrich Hansen

**Affiliations:** 1 Department of Mechanical Engineering, Imperial College London, South Kensington Campus, London SW7 2AZ, UK; j.clark14@imperial.ac.uk (J.N.C.); n.newell09@imperial.ac.uk (N.N.); 2 Department of Bioengineering, Imperial College London, London SW7 2AZ, UK; james.calder@fortiusclinic.com; 3 Fortius Clinic, 17 Fitzhardinge St, London W1H 6EQ, UK

**Keywords:** digital volume correlation, DVC, magnetic resonance imaging (MRI), ankle, bone, strain

## Abstract

Strains within bone play an important role in the remodelling process and the mechanisms of fracture. The ability to assess these strains in vivo can provide clinically relevant information regarding bone health, injury risk, and can also be used to optimise treatments. In vivo bone strains have been investigated using multiple experimental techniques, but none have quantified 3D strains using non-invasive techniques. Digital volume correlation based on clinical MRI (DVC-MRI) is a non-invasive technique that has the potential to achieve this. However, before it can be implemented, uncertainties associated with the measurements must be quantified. Here, DVC-MRI was evaluated to assess its potential to measure in vivo strains in the talus. A zero-strain test (two repeated unloaded scans) was conducted using three MRI sequences, and three DVC approaches to quantify errors and to establish optimal settings. With optimal settings, strains could be measured with a precision of 200 με and accuracy of 480 με for a spatial resolution of 7.5 mm, and a precision of 133 με and accuracy of 251 με for a spatial resolution of 10 mm. These results demonstrate that this technique has the potential to measure relevant levels of in vivo bone strain and to be used for a range of clinical applications.

## 1. Introduction

Understanding the mechanics of bone is a key factor to evaluating the (re)modelling process [1], preventing and treatment of fractures [2], and improving orthopaedic implant designs [3]. At the age of 60 years, 44% of women and 25% of men will experience a bone fracture at some point in the rest of their life [4], significantly increasing mortality rates even in relatively healthy patients [5]. The measurement of bone mineral density (BMD) is the most common clinical tool to assess the bone quality. Low BMD (i.e., BMD T-score ≤ −2.5) is a major risk factor for fractures, and it has been suggested that individuals with T-scores lower than −2.5 should be treated to decrease their risk of fractures [6,7]. However, solely using BMD as a factor to identify high-risk patients may not be accurate enough [8,9,10] as more than 50% of women and 70% of men do not have osteoporotic BMD levels (T-score ≤ −2.5) at the time of fracture [11]. The use of additional factors could improve evaluations of bone health.

Quantitative measurements of deformations and strains can significantly improve our knowledge and understanding of tissue health of musculoskeletal organs, which serve the purpose to generate, transmit and receive loads. Bone strain is the major stimuli for the bone remodelling process, which is required for maintenance of bone structure and strength [12]. Additionally, bone strain is directly related to both micro-crack formation [13,14] and the mechanism of bone fracture [2,15]. Therefore, strain may serve as a functional biomechanical marker that may be able to be used to evaluate bone health.

In the past decades, bone strains have been measured experimentally using contact techniques such as strain gauges (SG) and fibre Bragg grating sensors (FBGS), and non-contact techniques such as digital image correlation (DIC) and digital volume correlation (DVC) [16]. SGs have been commonly used for ex vivo [17,18] and in vivo [19,20] strain measurements in bone, and FBGS have been used for ex vivo strain measurements both within bone [21] and at bone-implant interfaces [22]. However, SG and FBGS are invasive techniques that are limited to measure strains at discrete locations only on the bone surface, and due to their non-negligible stiffness, they may reinforce the bone that they are attached to [23]. DIC is a non-contact technique that can provide full-field strain map of the bone surface. Although DIC has been extensively used for ex vivo studies [24], it is limited to surface measurements and since the bone surface needs to be exposed it is not a practical technique for in vivo strain measurement.

DVC is a powerful tool for calculating 3D internal strains in biological tissues, and has been used in combination with different imaging modalities such as micro computed tomography (microCT) [25,26,27,28,29,30,31,32,33,34,35], synchrotron radiation microCT (SR-microCT) [13,36,37], clinical CT [15,38], and micro magnetic resonance imaging (microMRI) [39] to quantify strains in bone, bone–cartilage, and at bone-biomaterial interfaces. Recently, DVC has been successfully used with weight-bearing clinical CT for in vivo quantification of the kinematics of the subtalar joint [40], demonstrating its potential for clinical use. However, the translation of microCT, SR-microCT, and microMR imaging-based DVC is limited to ex vivo use in human subjects. Additionally, the radiation associated with clinical CT is a limitation that reduces its suitability for use on patients.

Bone strain measurement using DVC in combination with clinical MRI has the potential to be a non-invasive technique to measure internal mechanical behaviour of human bone in vivo. When exploring new applications of DVC, it is important to evaluate the uncertainties associated with the technique. A zero-strain test is a widely used technique to quantify uncertainties of DVC measured displacements and strains on an object that has been strained a known amount (zero-strain) [26,28,32,33,37,38,41,42,43,44]. Therefore, the main aim of this study was to use DVC in combination with 3T clinical MRI for the first time to assess its potential to evaluate deformations and strains in human bones in vivo. The talus was used in this study to demonstrate the capabilities of the technique on a relevant bone. The talus is a high-risk stress fracture bone [45,46], and often exhibits osteochondral lesions and arthritis [47]. Therefore, identifying strain in the talus could help clinicians to define optimal patient-specific treatments. An additional objective of this study was to optimise the imaging and DVC settings to increase the accuracy and precision of DVC outcomes.

## 2. Materials and Methods

Three healthy asymptomatic male volunteers (age ranging 25 to 30 years old) participated in this study. The study was conducted in accordance with the Declaration of Helsinki, and the study received ethical approval from the Imperial College Research Ethics Committee (ICREC reference: 17IC4131). Written informed consent was obtained from all participants prior to imaging.

### 2.1. MRI Imaging and Optimisation of the Imaging Settings

**Figure 1:**
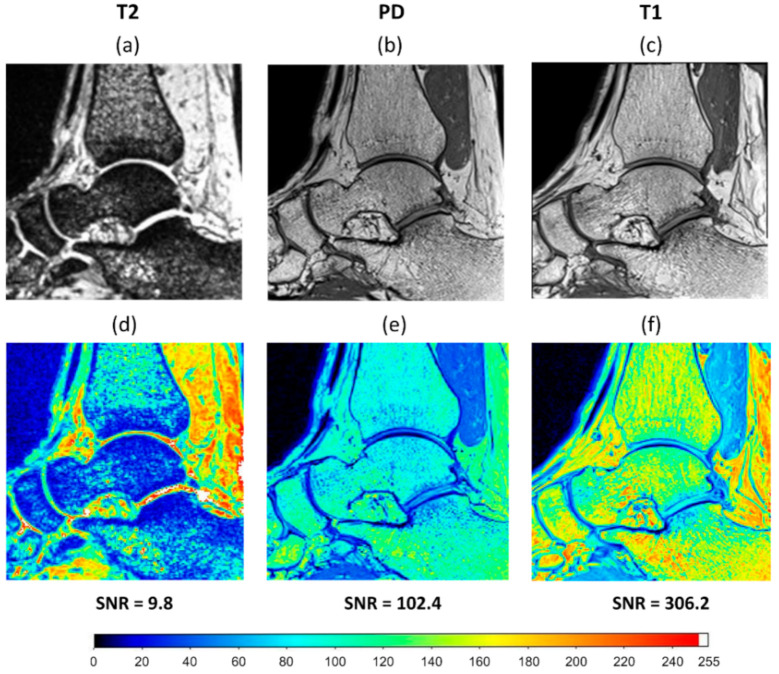
A sagittal slice of the clinical MRI of an ankle taken using three different MRI sequences: (**a**) T2 weighted; (**b**) proton density (PD) weighted; (**c**) T1 weighted. Signal intensity map (ranged from 0 to 255) and signal to noise ratio (SNR) achieved for (**d**) T2 weighted, (**e**) PD weighted, and (**f**) T1 weighted sequences.

MR imaging was performed using a 3T Siemens Magnetom Spectra (Siemens Medical Solution, Erlangen, Germany) with a standard 16 channel high-resolution ankle coil (Siemens Medical Solution, Erlangen, Germany). In order to find an optimal MR sequence to minimise DVC measurement uncertainties, three standard sequences were implemented: T2 weighted, proton density weighted (PD), and T1 weighted. 3D fast spin-echo (SE) acquisition mode was used for all sequences to obtain high-resolution 3D isotropic voxels. Details of each sequence parameters are reported in Table 1. Two consecutive zero-strain (i.e., unloaded) clinical MR images were captured for conducting each zero-strain test. To quantify DVC errors associated with the MRI sequences, a zero-strain test was initially conducted on the left ankle of only one volunteer using the three different sequences (Figure 1). Based on the DVC measured errors, the optimal MRI sequence (lowest errors) was selected. The sequence was then used to capture two unloaded scans (zero-strain) of the left ankle of all volunteers to investigate the repeatability of the strain measurement technique and to identify the optimal DVC settings. To minimise rigid body movement between the two scans, key anatomic features of the foot were aligned using the laser guidance system that was built into the MRI machine.

**Figure 2:**
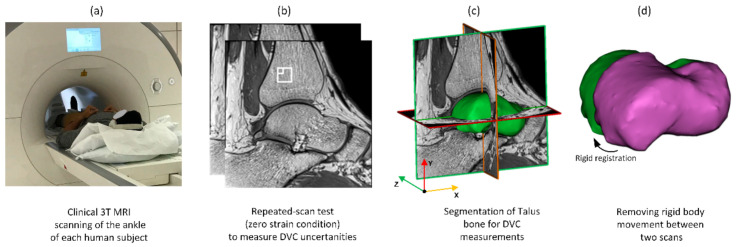
Workflow of the imaging and image post-processing: (**a**) MR images were obtained from each subject using a high-resolution standard ankle coil; (**b**) unloaded repeated scans captured from each ankle for the zero-strain test. The small and large white boxes correspond to subset sizes of 16 and 40 voxels, respectively; (**c**) segmentation of the talus bone to exclude surrounding tissues from DVC calculation; (**d**) registration to account for rigid body movement between scans.

Image post-processing was conducted before DVC analysis according to a fixed workflow (Figure 2). Each 3D image data set consisted of images including the distal tibia, talus, navicular, and calcaneus (Figure 2b) with 16-bit grey levels. The talus bone was selected for DVC analysis. Initially, sample specific 3D binary region-of-interest masks were created (Figure 2c) for each sample separately by manual segmentation of the talus using Mimics (Materialise HQ, v.19.0, Leuven, Belgium). These masks were used to exclude surrounding tissues (bone and other soft tissues) from the analysis, ensuring that only the talus tissue was analysed. Rigid registration was conducted (Fiji 1.53c, National Health Institute, Bethesda, MD, USA [48]) based on an iterative, multivariate, optimisation algorithm [49] that minimises the Euclidean distance (corresponding to the square root of summed squares of voxel intensity differences) between two unloaded scans. The calculated transformation matrix (three translational and three rotational parameters) between two scans was then applied to the target image to correct rigid body movement (Figure 2d). Finally, a left-handed orthogonal coordinate system was created on the posterior-distal corner of the first medial slice. The positive x-direction was directed anteriorly, the positive y-direction proximally, and the positive z-direction laterally (Figure 2c).

### 2.2. Digital Volume Correlation

DVC was conducted using DaVis 8.4 software (LaVision Ltd., Goettingen, Germany). To optimise DVC settings, three DVC algorithms were implemented in this study: Fast Fourier Transform (FFT) [15,26], Direct Correlation (DC) [27,28,32], and a combination of FFT and DC (FFT + DC) [42,43,44,50]. In all algorithms, the measurement volume was divided into smaller sub-volumes (called subsets) and the contrast patterns within the subsets were then tracked from the first volume (reference) into the second volume [51]. The displacement vectors for each subset were calculated to create the displacement field, and then strain components were calculated using displacement vectors. Further details of all approaches are explained in the following sections:

#### 2.2.1. DC Approach

The DC method involves tracking grayscale values of each subset from the undeformed images into the deformed images by shifting each subset in all directions and assessing the match between patterns. The quality of match was described by the correlation coefficient (*C*), which is maximal for a perfect match. The normalised correlation coefficient (*C_norm_*) was used to eliminate the effect of illumination changes between two scans. *C_norm_* for two volumes *A* (reference) and *B* (deformed) with shifts *dx, dy, dz* and window size of N × N × N voxels at point $\left(x_{0},y_{0},z_{0}\right)$ was computed according to:

(1) $$C_{norm}\left(dx,dy,dz\right)\;=\underset{\left(i,j,k\right)}{\sum }\frac{\left(A_{i,j,k\;}^{*})\;(B_{i+dx,j+dy,k+dz}^{*}\right)}{\sqrt[{2}]{{\left|A_{i,j,k}^{*}\right|}^{2}}\sqrt{{\left|B_{i+dx,j+dy,k+dz}^{*}\right|}^{2}}}$$

with

(2) $$A_{i,j,k}^{*}=A_{i,j,k}-\underset{\left(i,j,k\right)}{\sum }\frac{A_{i,j,k}}{N^{3}}$$

(3) $$B_{i+dx,j+dy,k+dz}^{*}=B_{i+dx,j+dy,k+dz}-\underset{\left(i,j,k\right)}{\sum }\frac{B_{i+dx,j+dy,k+dz}}{N^{3}}$$

(4) $${\left|A_{i,j,k}^{*}\right|}^{2}=\underset{\left(i,j,k\right)}{\sum }{\left[A_{i,j,k}^{*}\right]}^{2}$$

(5) $${\left|B_{i+dx,j+dy,k+dz}^{*}\right|}^{2}=\underset{\left(i,j,k\right)}{\sum }{\left[B_{i+dx,j+dy,k+dz}^{*}\right]}^{2}$$

In Equation (1) to (5) all summations are starting at $\left(i,j,k\right)\;=\;\left(x_{0},y_{0},z_{0}\right)$ and ending with $\left(i,j,k\right)\;=\;\left(x_{0}+N-1,y_{0}+N-1,z_{0}+N-1\right)$. Here $A_{i,j,k}$ is the intensity of the voxel at position $\left(i,j,k\right)$ in the volume *A* and $B_{i+dx,j+dy,k+dz}$ is the intensity of the voxel at the shifted position $\left(i+dx,j+dy,k+dz\right)$ in the volume B. The maximum of *C_norm_* corresponds to the shifts *dx*, *dy*, *dz* required to achieve the best match, and this shift is considered as the deformation vector of the subset.

#### 2.2.2. FFT Approach

In the FFT approach, rather than summing up all possible shift vectors for all subsets in all directions, the Fourier transform of the undeformed volume ($F\left(\mathrm{UD}\right)$) and the complex conjugate Fourier transform of the deformed volume ($F^{*}\left(D\right)$) are used. 3D correlation maps and displacement fields are obtained by calculating the inverse Fourier transform of $F\left(\mathrm{UD}\right)\;\times \;F^{*}\left(D\right)$. The advantage of using this method is that it provides a full correlation map for all possible shifts in the volume of interest with much less computational cost for large subset shifts compared to the DC approach.

#### 2.2.3. FFT + DC Approach

The third approach is a combination of the first two approaches (FFT + DC). This method uses the FFT approach for an initial prediction of large displacements, which are then refined using the DC approach. This approach was initially used to identify the optimal MRI sequence (subset size of 44 voxels), since previous studies [42,43,44] showed that FFT + DC has lower errors compared to the FFT and DC approaches for bone, cartilage and intervertebral discs. After identifying the optimal sequence, all DVC approaches were applied to it in order to quantify errors of each approach for different subset sizes.

### 2.3. Calculation of Strains from Displacements

DVC calculates one displacement vector for each subset, therefore spatial resolution is directly related to the number of subsets. From discrete displacement fields, strain tensor fields are quantified by the centred finite difference (CFD) scheme:

(6) $$\varepsilon_{ij}=\frac{1}{2}\left(\frac{\partial V_{i}}{\partial j}+\frac{\partial V_{j}}{\partial i}\right);\;\;i\in \left\{x,y,z\right\};\;\;j\in \left\{x,y,z\right\}$$

where $\varepsilon_{ij}$ represents the differences in displacements ($V_{i}$) between adjacent subsets along the j direction. As the displacement field is discrete:

(7) $$\varepsilon_{ij}=\frac{1}{2}\left(\frac{\partial V_{i}}{\partial j}+\frac{\partial V_{j}}{\partial i}\right)\;\approx \frac{1}{2}\left(\frac{\Delta V_{i}}{\Delta j}+\frac{\Delta V_{j}}{\Delta i}\right)$$

(8) Δi, Δj=2×subset length

### 2.4. Influence of Subset Size

Uncertainties associated with the DVC approach are influenced by the subset size [41,52]. Smaller subset sizes provide better spatial resolution to capture all features of interest, but they are more vulnerable to noise effects. Therefore, to evaluate the relationship between spatial resolutions and errors, DVC calculations were performed for seven subset sizes between 16 and 56 voxels. The multi-pass scheme is a predictor–corrector iterative technique adapted from particle image velocimetry (PIV) [53] that allows strains from the predictor step to be used to refine the proceeding corrector step. This technique has been used and explained in more detail in previous studies [37,42]. A multi-pass scheme with four steps (subset sizes: 64-56-48-40, number of iterations: 3) was used in this study to evaluate the effect of this technique on the errors of clinical MRI based DVC for strain measurement in bone. The minimal fraction of valid pixel (MFVP) [37] was set to 50% for all DVC calculations. Finally, a 50% subset overlap was used to increase the spatial density of vectors without decreasing the subset size for all approaches.

### 2.5. Quantification of DVC Uncertainties

Displacement and strain fields from the zero-strain test were extracted from DaVis and imported into a custom written MATLAB script to quantify displacement and strain errors (MathWorks, Inc, Natick, MA, USA). The methods used to calculate these errors are explained in the following sections.

#### 2.5.1. Displacement Errors

Since the real displacements of samples were unknown due to uncontrolled movements of subjects during scanning, random errors of displacements were used as a surrogate measure of displacement errors. Random errors were defined as the standard deviation (SD) of displacement components and were calculated for all three DVC approaches [33,36,51].

#### 2.5.2. Strain Errors

Any non-zero strains between the two sets of unloaded images were considered to be a result of strain measurement errors. Strain accuracy (MAER, Equation (9)), and precision (SDER, Equation (10)) were calculated as follows to provide a single strain error value to allow comparison with published literature:

(9) $$\mathrm{MAER}=\;\frac{1}{N}\overset{N}{\underset{k=1}{\sum }}\left(\frac{1}{6}\overset{6}{\underset{c=1}{\sum }}\left|\varepsilon_{c,k}\right|\right)$$

(10) $$\mathrm{SDER}=\;\sqrt{\frac{1}{N}\overset{N}{\underset{k=1}{\sum }}{\left(\frac{1}{6}\overset{6}{\underset{c=1}{\sum }}\left|\varepsilon_{c,k}\right|-MAER\right)}^{2}}$$

where N is the number of subsets, ε represents the strain, c represents each of six strain components, and k represents the measurement point [41]. To evaluate errors associated with each component of strain, in different sequences and approaches, accuracy (average of the absolute values of each component of strain) and precision (SD of the absolute values of each component of strain) were calculated and compared for each component of the strain separately [26,37].

## 3. Results

### 3.1. Influence of MRI Sequence

**Figure 3:**
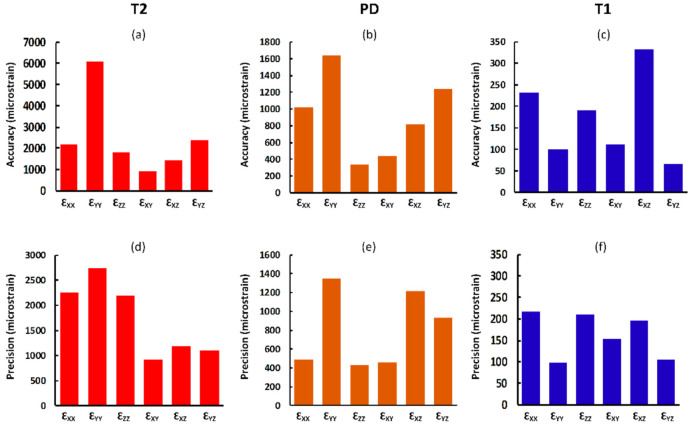
Analysis of the accuracy (**a**–**c**) and precision (**d**–**f**) of each component of strain for the T2, PD, and T1 sequences (subset size of 44 voxels). Due to the large differences in the values of errors for the three sequences, different scales were used.

Different MRI sequences resulted in different SNR and DVC uncertainties. SNR was highest for the T1 (306.2), followed by PD (102.4), and finally T2 (9.8) (Figure 1). Displacement random errors affecting all components of displacement, from each MRI sequence are reported in Table 2. They ranged between 6.0 µm and 10.3 µm for the T1, from 23.1 µm to 55.0 µm for the PD, and from 43.6 µm to 66.6 µm for the T2. Accuracy (MAER) and precision (SDER) errors were lowest for the T1 sequence (232 and 101 με, respectively), which were about four times lower than with PD (914 and 442 με, respectively) and ten times lower than T2 (2469 and 1245 με, respectively). To evaluate the range of errors and presence of preferential strain components in the sequences, accuracy and precision were reported for each component of strain separately (Figure 3). A similar trend to MAER and SDER was observed for each component of strain between the three MRI sequences (worst: T2; best: T1) for both accuracy and precision. The correlation coefficient also improved by more than 10% from T2 to T1 and reached a satisfactory value of 0.97 for T1.

### 3.2. Displacement Errors

The random errors affecting each component of displacement for the optimal sequence (T1) were measured using three different DVC computational approaches (Table 3). For the different subset sizes (from 16 to 56 voxels), the largest error was found for the DC approach (ranged between 9.5 and 46.5 μm), which was larger than those calculated with the FFT (ranged between 9.5 and 29.7 μm), and larger than those obtained with the FFT + DC (ranged between 2.9 and 25.4 μm). Random errors were larger for smaller subset sizes in all approaches, and the multi-pass scheme did not improve the outcomes of any of the approaches when compared to the results obtained with the single-pass scheme (subset size of 40 voxels).

### 3.3. Strain Accuracy (MAER) and Precision (SDER)

**Figure 4:**
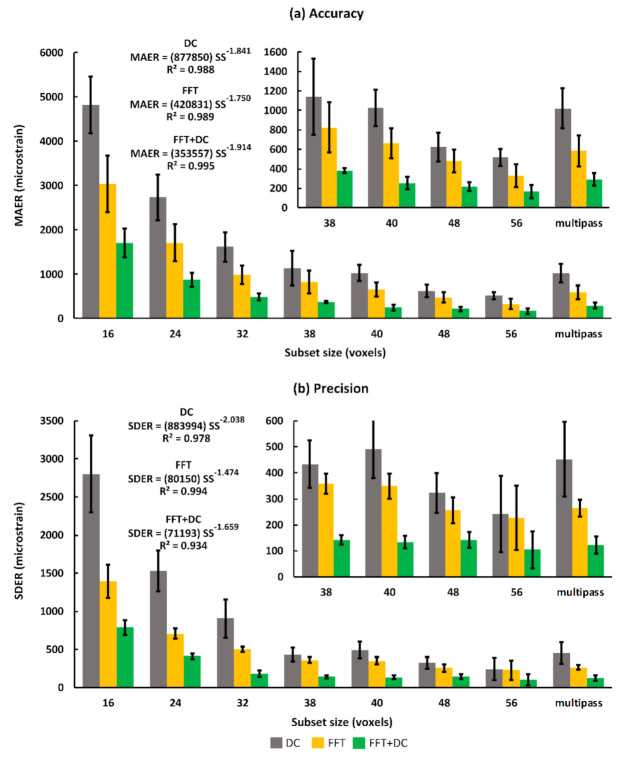
Trends of strain (**a**) accuracy (MAER) and (**b**) precision (SDER) as a function of subset size (voxels) for three DVC approaches applied to the T1 sequence. The trendline (power law) equations and R-squared values are reported for all three approaches. Bars and error bars represent mean and standard deviation (SD) values between the subjects. SS represents subset size in voxels in the equations reported.

Accuracy and precision were measured as a function of subset size to identify the relationship between strain errors and spatial resolution. As expected, the measured DVC accuracy and precision had decreasing trends with respect to the subset sizes for all computational approaches. As consistent with previous results in this study, for all subset sizes, accuracy and precision errors were lowest for the FFT + DC approach, followed by the FFT and the DC approach, respectively. In particular, for subset sizes ranging from 16 to 56 voxels, the mean values for MAER and SDER ranged between 168 με to 1707 με and 104 με to 789 με for the FFT + DC approach, between 331 με to 3038 με and 227 με to 1393 με for the FFT approach, and between 518 με to 4821 με and 243 με to 2804 με for the DC approach, respectively. A power law trend was observed between both MAER and SDER, and increasing subset size (SS in the equations in Figure 4). Power law relationships and coefficients of determination (R^2^) for all DVC approaches are reported in Figure 4. For the FFT approach, using the multi-pass scheme increased accuracy and precision by 11% and 24%, respectively (Figure 4), when compared to those obtained with the single-pass scheme (40 voxels). Conversely, the multi-pass scheme did not improve the outcomes of the DC and FFT + DC approaches at a similar subset size (40 voxels). For all subset sizes, and using all DVC approaches, the mean values of the C_norm_ were greater than 0.92.

### 3.4. Accuracy and Precision for Each Strain Component

**Figure 5:**
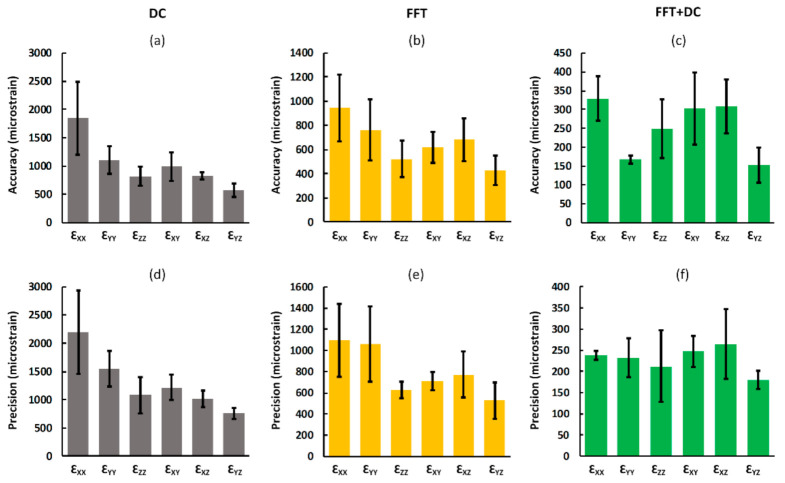
Analysis of the accuracy (**a**–**c**) and precision (**d**–**f**) of each component of strain for the DC, FFT, and FFT + DC approaches. For all DVC approaches the accuracy and precision of each component of strain was calculated as the mean and standard deviation, respectively, of the absolute values of each strain component between the subsets. Bars and error bars represent mean and standard deviation values between the subjects, respectively. Please note that due to the large differences in the values of errors for the three sequences, different scales were used.

For all DVC approaches, increasing the subset size improved the accuracy and precision of all strain components. Measured accuracy and precision of each strain component for a typical subset size (40 voxels) is shown in Figure 5. As observed from the displacement random errors, MAER, and SDER, when the individual components of strain were analysed separately, the lowest DVC uncertainties were obtained using the FFT + DC approach and highest uncertainties were associated with the DC approach (Figure 5). The differences between the maximum and minimum accuracy and precision of strain components were lowest for the FFT + DC approach (176 με and 85 με, respectively), followed by the FFT approach (515 με and 568 με, respectively), and then DC approach (1279 με and 1434 με, respectively).

### 3.5. Spatial Distribution of Errors

**Figure 6:**
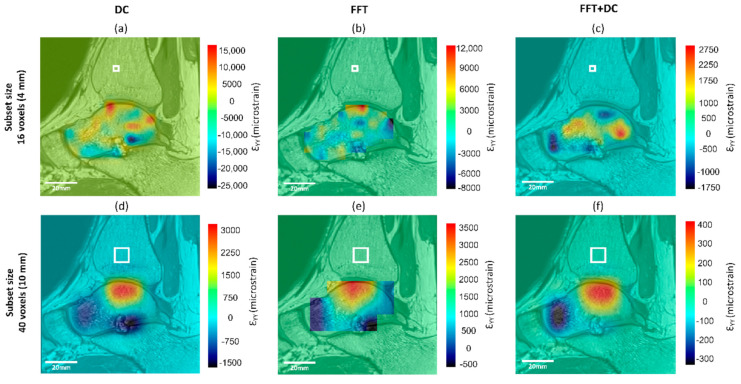
Spatial distribution of normal strain errors along the y-direction of the same sagittal slice for subset sizes of 16 (**a**–**c**) and 40 (**d**–**f**) voxels, calculated from the zero-strain test using the (**a**,**d**) DC, (**b**,**e**) FFT, and (**c**,**f**) FFT + DC approaches. White box in each strain map represents the subset size.

Generally, the patterns of spatial distribution of errors were similar for all DVC approaches with larger strain errors found close to the edge of the mask and the bone–cartilage interface. The distribution of the apparent normal strain along the y-direction (major load direction during standing) are shown for all DVC approaches and two subset sizes (16 and 40 voxels) for a typical subject in Figure 6. For a subset size of 16 voxels, the FFT + DC approach provided the lowest range of errors within the whole talus (between −337 με and 418 με), followed by the FFT (between −1231 με and 3651 με) and finally the DC approach (between −2951 με and 3278 με).

## 4. Discussion

The potential of DVC in combination with clinical MRI for strain measurement in bone was previously completely unexplored. For the first time in this study, the uncertainties of clinical MRI based DVC were evaluated in vivo using a zero-strain test. The aim of this study was to optimise imaging and DVC settings to improve its potential for clinical applications. Therefore, three different MRI sequences were investigated to identify the effect of imaging settings on the DVC outcomes and three different DVC approaches applied to the optimal sequence to evaluate strengths and limitations of each approach.

The findings reported in Table 2 indicate that using a T1 sequence can considerably improve DVC measurement uncertainties in comparison to PD and T2 sequences. For the same subset size, using a T1 sequence reduced the MAER and SDER up to five times, and random displacement errors up to four times compared to other sequences. This could be the result of higher SNR achieved using a T1 sequence that allows more features to be seen within the bone (Figure 1). Gilchrist et al. [54] also observed that higher SNR improve the performance of image registration algorithms, therefore SNR might be considered a contributing factor to image registration outcomes. The scanning time for the T1 sequence with the settings used in this study was less than four minutes, which makes it practical for simulating several physiological loading conditions during scanning. Moreover, it may be possible to reduce the errors further, either by using other MRI settings, or by enhancing images by filtering [37] which should be considered for future work.

The FFT + DC approach provided notably lower errors compared to the FFT and DC approaches (Figure 4), therefore the use of FFT + DC approach is recommended for clinical MRI based DVC strain measurement in bone. Moreover, based on zero-strain test results implementing a multi-pass scheme is not suggested for this application, as the multi-pass scheme did not improve the strain measurement errors, and even increased the errors by 15% compared to the single-pass scheme when using the FFT + DC approach. In line with previous DVC studies performed on microCT [26,32,43,44,55], SR-microCT [28,36], and microMRI [42], strain errors decreased with increasing subset size following a power law relationship. However, it should be noted that increasing the subset size reduces the spatial resolution. The equations presented in Figure 4 provided a framework to choose desirable DVC input parameters according to each specific application. In this study, the DVC errors for each individual component of strain was evaluated separately to investigate the presence of any preferential direction. The FFT + DC approach provided more isotropic behaviour compared to the FFT and DC approaches (Figure 5), with lower differences in the absolute magnitudes of errors between the strain components.

As has previously been observed from zero-strain tests in microCT and microMRI based DVC [32,42], maximum errors were found at areas with high voxel intensity gradients, which was at the bone–cartilage interface in this study (Figure 6). This was potentially caused by the significant changes in voxel grey intensity values at the transition from the bone to the other tissues (Figure 1). Smaller subset size resulted in the smaller areas in the talus affected by this edge effect, but it was observed for small subset size (16 voxels, Figure 6a–c) as well. Increasing the MFVP may improve errors at the edge of the mask, however, a larger MFVP would result in fewer calculated vectors, and therefore a progressive loss of data. Therefore, care must be taken when interpreting DVC outcomes in these areas and further work is required to reduce this edge effect.

Using the FFT + DC approach and subset sizes larger than 30 voxels (7.5 mm, provide 60 measurement points within the talus volume, Table 3) resulted in strain precision and accuracy errors consistently below 200 με and 500 με, respectively. Although the spatial resolution in this study is lower than ex vivo microCT based DVC (7.5 mm versus 0.5 to 2 mm) due to the lower resolution of clinical MRI scans (500 µm versus 10 to 40 µm), similar errors were achieved (SDER = 200 με and MAER = 300 to 650 με [26,28,32,33]) by optimising MR sequences and DVC settings. These errors are also comparable to errors associated with DIC techniques, which range from 100 με to 300 με [24,56]. Clinical CT-DVC has previously been used to evaluate femoral fractures ex vivo [15], with measurement errors in the range of 300 με to 500 με using a subset size 80% larger (~14 mm) than the subset size required to achieve this range of error in this study. Our study, by incorporating non-invasive non-ionising imaging modalities provides a progression of the DVC technique from the laboratory to the clinical setting. It should be noted that in vivo DVC presents some additional difficulties that can affect the DVC outcomes, such as moving artefacts, presence of other soft tissues and bones, and limitations in scan time. Due to these difficulties, a previous study [28] that used in vivo microCT images of anaesthetised mouse tibia’s was not able to obtain the SDER of 200 με since the typical diameter of the mouse bone was ~1000 µm which was approximately one third of the required subset size (~2800 µm) to reach the SDER of 200 με.

The errors found in this study represents 10% to 20% of the physiological strains (1000 με to 2500 με) seen on the trabecular bone measured in vivo using SGs on the surface of human cortical bone [19], and ex vivo using DIC [2,56]. The level of errors obtained in this study may be even lower than 10% to 20% of the physiological strains in certain bones that experience higher levels of strain during daily activities, such as the calcaneus. The calcaneus experienced notably higher strains (5500 με to 6000 με) during physiological activities (in vivo) compared with those of the medial tibia, lateral proximal femur, lamina of vertebra, and second metatarsal (393 με to 2301 με) [20]. Therefore, the errors in this study can be considered sufficient for in vivo strain measurement, given that a loading condition such as a single leg press generates a relatively high magnitude of bone strain [19].

Peña Fernández et al. [40] recently developed an in vivo technique using displacement fields extracted from DVC measurements based on weight-bearing clinical CT to quantify subtalar joint kinematics. They found displacement random errors ranging from 20 µm to 250 µm for weight-bearing clinical CT based DVC, which is larger than those obtained in this study for clinical MRI based DVC (maximum 30 µm). Therefore, clinical MRI based DVC may also allow the in vivo kinematics of joints to be quantified non-invasively. To identify failure mechanisms of human joints, clinical MRI-DVC can also be used for ex vivo studies to overcome the limitations of microCT, SR-microCT, and microMRI, which are all constrained by destructive sample dissections and sample-size restrictions [13]. The clinical MRI based DVC errors are lower than 6% of the yield values of bone tissue (10400 ± 1500 με [57,58]). Hence, its uncertainty is sufficient for ex vivo study of the fracture mechanism of intact, untreated joints in a more realistic condition.

A limitation of this study was that the DVC uncertainties were calculated in a homogeneous zero-strain condition provided by repeated scans. Zero-strain tests can be used to investigate the noise within a system and is an established method to quantify the DVC errors for different applications [26,28,32,33,37,38,41,42,43,44]. A zero-strain test also provides a standard protocol that allows a comparison of DVC uncertainties for different imaging modalities, mathematical approaches, and biological tissues. In addition, in vivo use of DVC in a strained condition can resulted in additional errors due to the extra moving artefact caused by non-uniformity of the applied load and more patient movements. Therefore, to obtain more accurate evaluation of DVC uncertainties future work is needed to calculate errors within strained bones.

In conclusion, this is the first study to use DVC in combination with in vivo clinical MR images to quantify the accuracy and precision of internal strain and displacement measurements in bone. We have shown the importance of optimising both imaging sequences and DVC settings for improving the accuracy and precision of DVC measurements. The optimised settings to measure in vivo bone strains were found to be a T1 MRI sequence (using parameters suggested in Table 1), with a single-pass FFT + DC DVC approach. This study has shown that clinical MRI based DVC can be used to measure in vivo bone strains under physiologic loads but can also be used to quantify in vivo joint kinematics and ex vivo evaluation of human joint fractures.

## Figures and Tables

**Table 1:** MR imaging sequences (T2 weighted, proton density (PD) weighted, and T1 weighted) parameters.

Sequences	Voxel Size (mm^3^)	TR ^1^ (ms)	TE ^2^ (ms)	ETL ^3^	BW ^4^ (Hz/px)	IG ^5^ (%)	FA ^6^ (°)	EN ^7^	NA ^8^	Scan Time (min:s)
T2	0.6 × 0.6 × 0.6	42	18	3	385	0	120	1	1	7:39
PD	0.5 × 0.5 × 0.5	900	30	37	355	0	120	1	1	5:23
T1	0.5 × 0.5 × 0.5	700	12	25	385	0	120	1	1	3:31

^1^ TR—repetition time, ^2^ TE—echo time, ^3^ ETL—echo train length, ^4^ BW—bandwidth, ^5^ IG—interstice gap, ^6^ FA—flip angle, ^7^ EN—echo numbers, ^8^ NA—number of averages.

**Table 2:** Signal to noise ratio (SNR), normalised correlation coefficient (C_norm_), Random errors of the computed components of displacement, and strain uncertainties (MAER and SDER) for the T2, PD, and T1 sequences (subset size of 44 voxels).

Sequence	SNR	Cnorm	Displacement Random Errors (µm)			Strain Uncertainties (µε)	
			x	y	z	MAER	SDER
T2	9.8	0.88	43.6	66.6	57.8	2469	1245
PD	102.4	0.95	32.2	23.1	55.0	914	442
T1	306.2	0.97	6.0	6.5	10.3	232	101

**Table 3:** Number of subsets (number of measurement points within the volume of the talus) and random errors of the computed components of displacement for the Fast Fourier Transform (FFT), Direct Correlation (DC), and FFT + DC DVC approaches for different subset sizes (16 to 56 voxels) and the multi-pass (MP) scheme (final subset size of 40 voxels). Median values are reported.

Subset Size (voxel-mm)	NS ^1^	FFT			DC			FFT + DC		
		x (µm)	y (µm)	z (µm)	x (µm)	y (µm)	z (µm)	x (µm)	y (µm)	z (µm)
16–4	462	26.5	29.7	25.7	46.3	46.5	30.4	25.4	24.7	19.1
24–6	129	19.2	24.0	21.5	37.4	37.8	24.1	17.1	16.3	15.9
32–8	61	18.1	21.2	19.4	32.6	29.2	21.4	13.8	11.6	14.7
38–9.5	37	14.7	16.1	17.4	27.9	20.2	17.3	13.7	14.2	12.7
40–10	27	15.6	18.8	19.2	28.6	20.0	20.6	7.1	12.1	12.3
48–12	13	13.9	11.0	18.5	18.4	15.7	18.7	7.6	7.8	13.6
56–14	8	9.5	10.6	13.4	14.4	9.5	15.1	9.3	2.9	7.7
MP ^2^	27	16.5	20.7	11.9	31.7	21.3	17.4	15.1	13.7	10.5

^1^ NS number of subsets; ^2^ MP multi-pass.
